# Tracking and Comparing Self-Determined Motivation in Elite Youth Soccer: Influence of Developmental Activities, Age, and Skill

**DOI:** 10.3389/fpsyg.2019.00304

**Published:** 2019-03-05

**Authors:** David T. Hendry, Peter R. E. Crocker, A. Mark Williams, Nicola J. Hodges

**Affiliations:** ^1^Department of Sport, Exercise and Rehabilitation, Northumbria University, Newcastle upon Tyne, United Kingdom; ^2^School of Kinesiology, The University of British Columbia, Vancouver, BC, Canada; ^3^Health, Kinesiology, and Recreation, The University of Utah, Salt Lake City UT, United States

**Keywords:** expertise, sports, talent identification, deliberate practice, play

## Abstract

**Purpose:** Our aim was to determine if self-determined motivation (SDM) in elite, men’s soccer changes over time and differs as a function of age, skill-grouping, and engagement in soccer play and practice. We tested predictions from the Developmental Model of Sport Participation (DMSP) regarding relations between practice and play and SDM among both elite and non-elite samples.

**Methods:** Elite youth soccer players in the United Kingdom (*n* = 31; from the Under 13/U13 and U15 years age groups) completed practice history and motivation questionnaires at time 1 (T1) and ∼2 years later (T2: now U15 and U17 years). Non-elite players (*n* = 32; from U15 and U17 years) completed the same questionnaires at T2 only.

**Results:** For the elite groups, global SDM decreased over time for the current U17 group (from U15), but no change was seen for the current U15 group (from U13). Age group differences at T2 mirrored these data, with U17 players showing lower global SDM and higher controlled motivation than U15 elites. The non-elite players did not show age group differences, but elites scored higher for global SDM and autonomous motivation than non-elites. The recent hours accumulated in practice negatively correlated with global SDM in elite and non-elite groups, but play was unrelated to measures of motivation.

**Conclusion:** Differences in SDM as a function of age and skill point toward the dynamic nature of these motivations over time, likely a result of proximity to external rewards related to professional status. Although high volumes of practice are related to lower global SDM in both skill groups, the absence of any relations between SDM and soccer play does not support a key prediction related to the DMSP.

## Introduction

A multitude of psychological characteristics potentially influence the pathway toward expertise in sports (e.g., Jordet, 2015). Motivation is considered an essential characteristic of expertise, since high levels of motivation are considered necessary to sustain time and effort in activities aimed at improving performance. Numerous published reports have highlighted emerging ideas and evidence that either purport to or show relationships between developmental activities (practice and play) and motivation (e.g., Côté et al., 2012; Hendry et al., 2014; Vink et al., 2015). In addition to studying relations between self-determined motivation (SDM, e.g., Ryan and Deci, 2017) and accumulated hours in various developmental soccer activities among elite and sub-elite male youth soccer players, we assess if and how these motivations change over time and covary with expertise.

Numerous talent development programs select aspiring experts at increasingly younger ages, with a view to optimizing the volume and quality of practice (Côté et al., 2011). Yet, the overall efficacy of this early selection approach and its psychosocial impact on players has been questioned (e.g., Côté and Erickson, 2015). There is evidence that “deliberate play” activities (i.e., unorganized, self-led, sporting activities that are not conducted with a coach/teacher) during childhood can contribute to the emergence of adult expertise and foster positive forms of motivation (e.g., Berry et al., 2008). These findings are encapsulated within the Developmental Model of Sport Participation (DMSP; Côté et al., 2007; Côté, 1999).

The DMSP consists of two primary pathways toward sports expertise; one based on early specialization and deliberate practice in one sport from an early age and a second involving sampling of different sports and play-based sporting activities during childhood and later specialization. The early specialization pathway is based on ideas emanating from the deliberate practice framework and the assumption that a monotonic relationship exists between deliberate practice activities, engaged in with the primary intent of improvement, and performance (Ericsson et al., 1993). According to the DMSP, sport expertise might also be served by a second “sampling and play” pathway. This second pathway is thought to circumvent the potentially negative consequences associated with early specialization, such as increased incidence of burnout, drop-out, injury and a general decline in well-being (e.g., Côté et al., 2007). The largely volitional and enjoyable nature of deliberate play in childhood is thought to develop intrinsic and self-determined forms of motivation that facilitate long-term sport participation (e.g., Côté et al., 2007, 2012).

There is a considerable body of evidence in sport supporting the idea that skill and deliberate (or purposeful) practice are positively related and hence high volumes of deliberate practice are needed to succeed (see Ford et al., 2015). As learners must invest maximal cognitive and physical effort over an extended period of time in deliberate practice, motivation is central to this framework (Ericsson and Towne, 2010). Different types of motivation are required to engage in deliberate practice activities since these activities are often described as not always being inherently enjoyable (e.g., Ericsson et al., 1993). Furthermore, the reasons for engaging in deliberate practice may change from engaging in practice for enjoyment in practice itself (i.e., intrinsic motivation), to enjoyment from the rewards of practice, such as improved performance and success, Ward et al., 2007).

The complex nature of motivation and its role in practice engagement is encompassed within Self-Determination Theory (SDT; e.g., Ryan and Deci, 2017). SDT is a meta-theoretical framework which offers a nuanced, multidimensional account of motivation. At the forefront of this theory is the idea that humans have an innate tendency to seek growth and embrace challenges which results in engagement in an activity for interest and enjoyment (i.e., intrinsic motivation). Central to SDT is Organismic Integration Theory (OIT; Ryan and Deci, 2017). The OIT places motivation along a continuum of self-determination, in which initial engagement in an activity for contingent (or externally rewarding) reasons can become internalized over time. As such, behavior becomes progressively integrated into one’s sense of self (i.e., more self-determined). There are three broad types of motivation, namely, intrinsic, extrinsic, and amotivation, which are underpinned by six behavioral regulations. Intrinsic regulation (IM) occurs when an individual performs for enjoyment or interest. Next on the continuum is extrinsic motivation (EM), consisting of four behavioral regulations. As the most self-determined motivation, integrated regulation (IG) reflects a full assimilation of the values and beliefs from the activity into a sense of self. The individual participates in sport because they identify themselves as an athlete and live their life in accordance with becoming a better athlete (Taylor, 2015). Identified regulation (ID) signifies sport engagement because the benefits of sport involvement are highly valued. Participating in sport to avoid feelings of shame or guilt associated with non-participation is referred to as introjected regulation (IJ). These feelings may occur when an athlete participates to appease family members or feelings of contingent self-worth. External regulation (EM), which signifies sport involvement to seek rewards (e.g., trophies or medals) or avoid punishment (scolding from parents/coaches) is the least self-determined EM. Amotivation (AM) denotes a complete lack of motivation. Behavioral regulations can be encompassed within two higher order themes: autonomous (including intrinsic, integrated and IDs); and controlled motivation (including introjected and external regulations). Generally speaking, autonomous forms are associated with positive outcomes, whereas controlled motivation are largely related to negative outcome (Ryan and Deci, 2017).

According to Côté and colleagues, the largely volitional and enjoyable nature of deliberate play in childhood should develop intrinsic and self-determined forms of motivation (e.g., Côté et al., 2007, 2012). This suggestion is in contrast to deliberate practice, which is often externally controlled, at least in sports, and not necessarily intrinsically rewarding. Regardless, in a study of three groups of elite, youth soccer players (ages Under 13 years/U13, U15, and U17 years), there were no associations between accumulated hours in childhood, play-type activities and measures of SDM for any of the age-groups (Hendry et al., 2014). However, for the oldest group of soccer players (i.e., U17), accumulated hours in Academy practice were negatively related to global measures of SDM and positively related to controlled motivation. This U17 age group was shown to be less autonomously motivated than the younger age-groups (U13 and U15) and had lower scores for integrated and identified behavioral regulations, suggesting a diminished value of soccer and a reduced assimilation between the game and their sense of self.

Organismic Integration Theory offers some means of understanding the complexity of motives for athletes and may aid our understanding of the relationships between early sport activities in developing SDM. According to this theory, changes in SDM are moderated by several factors such as external rewards, age and skill. In a meta-analytic review of SDM in educational contexts, the use of external rewards was shown to undermine autonomous motivation (Deci et al., 2001). Although external rewards typify the attainment of professional status in many sports, in particular men’s soccer, changes in SDM over time, as the lure of professional rewards become more salient, has not to date been investigated in longitudinal-type investigations.

Age-related declines in SDM have been shown in non-elite, physical education settings during early adolescence, perhaps related to competing interests at this age (12–14 years; e.g., Otis et al., 2005; Barkoukis et al., 2014). However, higher performing students did not show this decline. A positive association was seen between students’ performance and autonomous (or self-determined) motivation (Barkoukis et al., 2014). Based on these factors, there is reason to suspect that SDM would change over time, potentially as a function of age and skill, becoming less autonomous with age (around adolescence) and then later more autonomous as skill is achieved. In high-level, youth sports, where the lure of external rewards increase with age, there is reason to suspect that motivations would become less rather than more autonomous.

According to Taylor (2015), controlled forms of motivation related to performance improvement, achieving status positions and winning competitions, become increasingly important through the transitions toward adult expertise. Aspects of controlled motivation, such as introjected regulation, appear to facilitate perseverance and resilience, which are needed when practice or competition become demanding and/or monotonous (Gillet et al., 2009, 2012; Hardy et al., 2017).

In the current study, we followed up elite-soccer players who had progressed from U13 to U15 (years) and from U15 to U17 (years), soccer-Academy age groups. We compared the current U15 and U17 elite-age groups with age-matched non-elite soccer groups, to assess whether any age-related differences in SDM were indicative of general developmental trends in sports, unrelated to the elite-Academy setting. We expected to see a general reduction in autonomous motivation with age (from U15 to U17 year, but not from U13 to U15 years) and an increase in controlled motivation, yet we were unsure the extent to which these declines would covary with skill. Although there was reason to suspect declines in measures of SDM in adolescence (e.g., Otis et al., 2005; Barkoukis et al., 2014), the nature of external rewards associated with professional contracts as the elite-youth players progress from U15 to U17 years, might lead to the prediction that age group differences will be specific to elite groups.

A second reason why age-group differences or declines in measures of SDM might be observed in older groups of youth-elite soccer players is related to the quantity and demands of practice. Therefore, we evaluated whether engagement in recent soccer practice and play amounts (i.e., over the 2.5 years period where they were prospectively tracked) was related to current measures of motivation and any changes in motivation over this time period (see, Côté et al., 2012). We expected that more time spent in formal practice across the intervening years would be negatively related to autonomous, and positively related to controlled motivation. Based on earlier research (Hendry et al., 2014), we did not expect relations between play and motivations, at least for the elite sample. For the non-elite group, childhood play may be an important variable in promoting long term self-determined motivation, because the relative amounts of play versus practice are expected to be larger and other factors related to extrinsic rewards are less likely to moderate any potential relationships. For this non-elite group, we assessed accumulated practice and play in childhood as well as in the more recent years.

## Materials and Methods

### Participants

We collected data from 63 male, youth soccer player (*n* = 31, elite players from five professional youth Academies in Scotland; *n* = 32 non-elite players from Western Canada). The elite players completed practice and motivation questionnaires at T1 (October 2011; see Hendry et al., 2014) and T2 (January 2014). The elite players, participating in the highest tier of Scottish youth soccer, had transitioned through their respective professional soccer academies from U13 (12–13 years) and U15 (14–15 years) at T1 to U15 (*n* = 15) and U17 (*n* = 16; 16–17 years) in the longitudinal follow-up (T2). Data from the non-elite group were collected from U15 (*n* = 16) and U17 (*n* = 16) age-group players, playing in third tier of competitive youth soccer at the regional/local level in Western Canada (December 2015). According to Baker and colleagues’ taxonomy (Baker et al., 2015), these groups would be classified as advanced/expert (elite) and basic (non-elite), youth sport athletes. There were no significant age differences between the elite and non-elite groups for either the U15 (*t*(29) = 1.71, *p* = 0.09, *d* = 0.25) or U17 age groups (*t*(29) = 0.16, *p* = 0.88, *d* = 0.04). Both the elite and non-elite groups, whilst different to each other, had accumulated a similar number of soccer activity hours (including match play) as detailed in previous studies of soccer players participating in the United Kingdom (∼3000–5500 hr; e.g., Ford and Williams, 2012; see also Table 1).

**Table 1:** Means, (SDs) and 95% confidence intervals corresponding to accumulated and weekly hours in practice and play (during childhood and across the player’s careers) for the elite and non-elite groups, as well as start age in soccer activities and number of sports participated in childhood.

Soccer activity and age	Elite	Non-elite	t	Cohen’s d	95% CI (mean differences)	
	*n* = 31	*n* = 32				
	(U15 = 15; U17 = 16)	(U15 = 16; U17 = 16)			Lower	Upper
**Childhood (5–12 years; hrs):**						
Accumulated soccer practice	1834 (824)	886 (367)	6.35^∗∗^	1.55	629.25	1266.75
Accumulated soccer play	2259 (1156)	888 (608)	4.21^∗∗^	1.04	909.45	1832.55
**Career (5 years – current years; hrs):**						
Accumulated soccer practice	2741 (1083)	1403 (466)	3.53^∗^	0.86	955.34	1720.73
Accumulated soccer play	2724 (887)	1224 (814)	9.81^∗∗^	2.42	895.58	1746.42
Current weekly soccer practice	8.29 (2.34)	3.07 (0.49)	16.16^∗∗^	3.69	4.37	6.06
Current weekly soccer play	3.91 (2.50)	2.14 (1.95)	3.24^∗∗^	0.79	0.06	2.89
**Recent soccer activities (last 2.5 years; hrs):**						
Accumulated soccer practice	907 (212.62)					
Accumulated soccer play	465 (324.30)					
**Soccer milestones:**						
Start age soccer (years)	4.55 (1.21)	5.24 (1.26)	2.46^∗^	0.56	0.07	1.31
Start age soccer practice (years)	5.80 (1.98)	6.44 (1.81)	1.49	0.03	-0.03	1.51
Number of other sports	2.61 (1.35)	4.44 (1.21)	6.29^∗∗^	1.43	1.18	2.47


*Statistical analyses are also presented based on independent *t*-tests (*df* = 61). Cohen’s *d* is given as a measure of effect size. ^∗^*p* < 0.01, ^∗∗^*p* < 0.001.*

T1 motivation scores from elite players in this study, were part of a larger sample reported in previous research (Hendry et al., 2014). The T1 scores were included within the current study as a means of assessing change in motivation from T1 to T2 within the same sample of players. The ∼2.5 year gap between data collection points corresponded to age-related differences based on cross-sectional comparisons observed in previous work. Parents were given three weeks to object from their child participating in the study, otherwise passive consent was assumed. On the day of data collection, players and a subsection of parents who completed the questionnaires for reliability purposes, provided written consent before completing the questionnaires. Players were under no obligation to complete the questionnaires and coaches were not made aware of who participated. Procedures were approved by Behavioral Research Ethics’ Board of the University of British Columbia.

### Procedures

**FIGURE 1:**
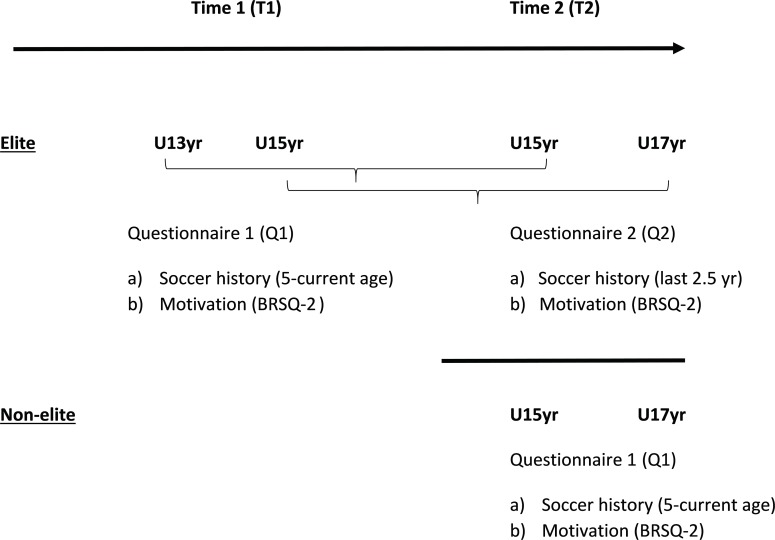
Schematic to show the chronology of our procedures for collecting soccer activity estimates and self-determined motivation scores from the elite and non-elite players at time 1 and time 2.

Initial recruitment was made via email correspondence with participating clubs. Figure 1 provides a schematic of the overall data collection procedure. At T1, elite players completed questionnaire 1 (Q1) which included a soccer-specific, practice history questionnaire and the Behavioral Regulation in Sport Questionnaire (BRSQ, Lonsdale et al., 2008). The data were collected in small groups supervised by the first author, such that clarification and assistance could be provided when needed. At T2, elite players completed questionnaire 2 (Q2), which included a truncated version of the soccer activity questionnaire focusing on the developmental activities engaged in between T1 and T2 (∼2.5 years period), as well as the full BRSQ. To aid convergent validity, a sample of parents (T1, *n* = 6; T2, *n* = 4) provided career estimates of soccer practice and play using the same questionnaire. Also, coaches (T1, *n* = 6; T2, *n* = 4) provided estimates of the number and content of a typical week’s organized practice session for their respective age groups (see Hopwood, 2015).

Non-elite players completed Q1 only and followed the same procedures as the elite group at T1. Players’ coaches (*n* = 5) provided estimates of the number and content of a typical week’s organized practice session and a sample of parents (*n* = 4) provided career estimates of hours in soccer activities. Participating clubs were contacted via email at T1 and T2 and follow-up emails and meetings were made with the individual team managers or coaches.

### Measures

#### Retrospective Questionnaires

The soccer-specific practice questionnaire was adapted from the “Participation History Questionnaire” (PHQ, e.g., Ford et al., 2010) and previous research related to testing of the deliberate practice framework [initially based on methods used by Ericsson et al. (1993)]. This questionnaire and similar versions have received validation with respect to their ability to provide estimates that differentiate across elite and less elite samples, matching of estimates across current weekly practice amounts, diary estimates and estimated yearly amounts, matching of estimates across coach, parent and athlete samples as well as validation from triangulation of retrospective methods with age-related, cross-sectional samples (e.g., Hodges and Starkes, 1996; Helsen et al., 1998; Hodges et al., 2004; Ward et al., 2007; Ford et al., 2009, 2010). This retrospective method remains the best available method for collecting practice histories from elite athletes (Hopwood, 2015).

Basic demographic information pertaining to start age in soccer activities, typical current weekly practice amounts in soccer (for reliability purposes), total number of other sports engaged in outside of school, and the number of years in the academy system were collected in Q1 and Q2. Operational definitions and examples of organized practice and play were provided. Practice was defined as activities conducted with a coach/adult used mainly to improve skills (i.e., formal practice). In this sense, organized practice provides a proxy measure of deliberate practice typically engaged in during formal/coach structured activity (e.g., Tedesqui and Young, 2017). Play was defined as unorganized, self-led activities that are not conducted with a coach/teacher (i.e., informal, self-led soccer activities). Players provided estimates of: (i) number of organized practice sessions/week; (ii) average duration of each session; and (iii) hours/week in soccer play, during a typical week. These data were solicited from 5 years of age to the present time in 2-year intervals (i.e., 5–6 years, 7–8 years …15–16 years). To estimate accumulated practice/play hours for years between each of these age-intervals, we took an average of the surrounding years (e.g., to estimate practice for 6–7 years, the average of hours reported for 5–6 years and 7–8 years was calculated). Significant breaks from soccer were recorded.

The hours accumulated in practice were calculated by multiplying hours per session by the number of sessions/week. This number was multiplied by the average reported season length for participating players, subtracting the number of weeks lost through illness or injury for individual players (which equated to an average of ∼46 weeks practice/year). This procedure was repeated for soccer play. We calculated accumulated hours in soccer practice and play during childhood (5–12 years) and across careers (5–current years). Questionnaire 2 (Q2) was a truncated version of Q1. Although it consisted of the same demographic and developmental soccer activity questions as in Q1, it differed in that data were collected every year for the 2.5 year period spanning T1 to T2.

In order to assess reliability and validity, intra-class correlations (*ICCs*) and percent agreement (*PA*, based on division of the smaller by the largest value for each pair, multiplied by 100) were calculated for: (i) the player-player estimates within the same questionnaire for Q1; (ii) player-player weekly estimates from Q1 (last years) and Q2 (first years); (iii) coach-player weekly estimates of soccer practice; and (iv) parent-player estimates of accumulated hours spent in developmental soccer activities (i.e., both practice and play). These give an indication of the strength of the relations and similarity between estimates, respectively. Such combined analyses have been recommended as the most comprehensive assessment of validity and reliability of activity estimates (Atkinson and Nevill, 1998; Hopwood, 2015). All reported analyses are unique, although the elite participants (data at T1 only), were part of a larger sample (*N* = 144) of elite youth athletes reported in Hendry et al. (2014).

##### Elite

At T1 (elite group only), the strength and similarity of player-player estimates of time spent in weekly soccer activities (from different sections of the questionnaire) were deemed moderate to high and increased for more recent estimates (*n* = 31; *PA* range = 68.1–83.4%, *ICC* range = 0.46–0.91, *p’s* < 0.05). In comparing the time-period during which estimates from Q1 and Q2 overlapped (elite group only), the strength and similarity was again high for estimates of play (*PA* = 83.5%, *ICC* = 0.87) and practice (*PA* = 93.1%, *ICC* = 0.91). Also, there was a high correlation (*ICC* = 0.92) and degree of similarity (*PA* = 91.3%) between coach and player estimates of weekly practice hours. Parent-player estimates (based on accumulated hours) were moderately correlated for both practice (*PA* = 59%, *ICC* = 0.58) and play (*PA* = 56%, *ICC* = 0.60). Similar reliability was established at T2 for the elite players. There was a high correlation (*ICC* = 0.94) and similarity (*PA* = 92.7%) between player and coach weekly practice estimates and between player-parent estimates for both practice (*PA* = 80.1%, *ICC* = 0.82) and play (*PA* = 75.6%, *ICC* = 0.73).

##### Non-elite

For the non-elite players, player and coach estimates of weekly practice fell within the high range (*PA* = 82%, *ICC* = 0.84), as did player and parent estimates of accumulated hours in play (*PA* = 70%, *ICC* = 0.76) and practice (*PA* = 85%, *ICC* = 0.90).

#### Motivation

The 24 item, BRSQ uses four item subscales to measure each of the six behavioral regulations from SDT and provides overall indices of motivation (see Table 2). Participants responded to the following stem; “I participate in soccer because…” before responding to each item using a 7-point Likert scale where *1 = not at all true, 4 = somewhat true and 7 = very true*. The items for each subscale were aggregated to provide an overall (average) score for each behavioral regulation. Global indices of SDM (SDI) and autonomous and controlled motivation were calculated by applying a coefficient to the behavioral regulations, see Table 2 (Hodge and Lonsdale, 2011). The reliability of each behavioral regulation score was determined using Cronbach’s α = 0.70 (*IM* = 0.73; *IG* = 0.72; *ID* = 0.74; *IJ* = 0.75; *EM* = 0.79; *AM* = 0.86). Given the low number of items used to measure each subscale, these values were deemed acceptable (Cortina, 1993). Motivation change scores were calculated for the elite players that had completed the BRSQ at T1 and T2. To ameliorate potential for Type 1 error, we focus primarily on composite scores of SDI (overall self-determined motivation index score) and autonomous and controlled motivation, given that these measures were most related to our predicted age and or skill group effects.

**Table 2:** Mean (and SD) self-determined motivation scores of the current U15 and U17 elite and non-elite soccer players at time 1 (T1) and time 2 (T2).

	Elite				Non-elite	
	U15		U17		U15 T2	U17 T2
	T1 (U13)	T2 (U15)	T1 (U15)	T2 (U17)		
**Motivation indices**						
SDI (Max = 25)						
(2 × IM + 1 × IG + 1 × ID +	16.00 (4.10)	18.41 (3.08)	16.42 (5.96)	13.03 (5.19)	13.08 (4.25)	12.89 (4.85)
(-1) × IJ + (-2) × EX))						
Autonomous EM (Max = 28) (2 × IM + 1 × IG + 1 × ID)	26.88 (1.00)	26.63 (1.34)	26.03 (1.92)	24.88 (1.60)	23.52 (2.94)	23.98 (2.83)
Controlled EM (Max = 21) (-1 × IJ + (-2) × EX)	10.88 (4.28)	7.63 (4.29)	9.55 (6.38)	11.86 (7.37)	10.44 (2.51)	10.19 (3.41)
**Behavioral Regulations (Max = 7)**						
Intrinsic (IM)	6.98 (0.75)	6.94 (0.14)	6.89 (0.30)	6.67 (0.40)	6.59 (0.46)	6.54 (0.63)
Integrated (IG)	6.76 (0.52)	6.62 (0.48)	6.35 (0.75)	6.07 (0.68)	5.36 (1.12)	4.94 (1.07)
Identified (ID)	6.15 (0.82)	6.14 (0.94)	5.89 (0.98)	5.54 (0.93)	4.97 (1.26)	5.06 (1.00)
Introjected (IJ)	3.74 (1.83)	2.55 (1.35)	2.90 (1.66)	3.75 (2.32)	3.17 (1.08)	3.01 (1.15)
External (EX)	1.70 (0.78)	1.32 (0.51)	1.86 (1.65)	2.18 (1.51)	2.05 (0.66)	2.08 (0.87)
Amotivation	1.38 (0.38)	1.04 (0.17)	1.36 (0.56)	1.86 (1.52)	1.63 (0.87)	1.36 (0.56)


*SDI = self determination index; IM = intrinsic motivation; EM = extrinsic motivation.*

### Statistical Analyses

The data were checked for normality using the Shapiro-Wilk test. When the magnitude of skewness was less than 1, indicating only a tendency toward positive skewness (Bulmer, 1979), and there were no significant differences in homogeneity of variance between the groups, we used parametric methods for our analyses based upon the robustness of this technique to violations to normality (Glass et al., 1972; Pallant, 2007). In cases where assumptions were not met, which was the case for accumulated soccer activity estimates, non-parametric tests were used to assess relationships (i.e., Spearman’s correlation coefficient). Confidence intervals (95%) around mean differences for significant pairwise comparisons and for Pearson’s correlations are provided. All statistical analyses were conducted using IBM SPSS version 22.

#### Soccer Development and Demographics

Independent *t*-tests were used to test differences between the elite and non-elite players with respect to various soccer-related demographics including: start age in soccer; start age in soccer practice; current age; number of other sports; and hours per week and accumulated hours in play and practice. For significant results Cohen’s *d* provided estimates of the effect size.

#### Motivation Comparison Across Age and Skill

As part of the prospective assessment of motivation for the elite group, we ran a 2 (Current Age category; U15 and U17 years) × 2 (Time; T1, T2) repeated measures ANOVA for the primary dependent variables, SDI, autonomous and controlled motivation. To determine whether any potential age-related differences in motivation were specific to the elite group, we conducted separate 2 (Skill level; Elite, Non-elite) × 2 (Current Age category; U15, U17) between-participants ANOVAs for the same indices of motivation as noted above and used Tukey HSD *post hoc* tests to evaluate interactions. Partial eta-squared (η_p_^2^) provided an effect size measure for between group comparisons and alpha was set at 0.05 for the testing of statistical significance.

#### Soccer Activity Relationships With Motivation

Spearman correlations indexed the relationships between indices of motivation and accumulated hours in soccer activities. For non-elite players, relationships between indices of motivation and accumulated childhood (5–12 years) soccer activities were assessed. For, elite players, indices of motivation at T2 were correlated with both childhood soccer activity and more “recent” activity occurring in the last 2.5 years (T1–T2). In order to potentially explain any change in motivation across time, we analyzed the relationship between change in indices of motivation (from T1 to T2; elites only) and recent practice over this same time period. Alpha (α) was set at 0.05 for all correlations with *r*_s_ > 0.30, considered to reflect a moderate effect size (Cohen, 1988).

## Results

### Soccer Development and Demographics

Table 1 shows the mean, soccer-related practice data and inferential statistics comparing the elite and non-elite groups. The elite players engaged in more soccer practice and play/week, accumulated more hours in soccer practice and play, engaged in general soccer activities earlier and participated in fewer sports when compared with the non-elite group (*p’s* < 0.05). The groups did not differ with respect to when they first participated in soccer practice.

### Motivation Comparisons Across Age, Time and Skill

#### Changes in Motivation Among Elites

**FIGURE 2:**
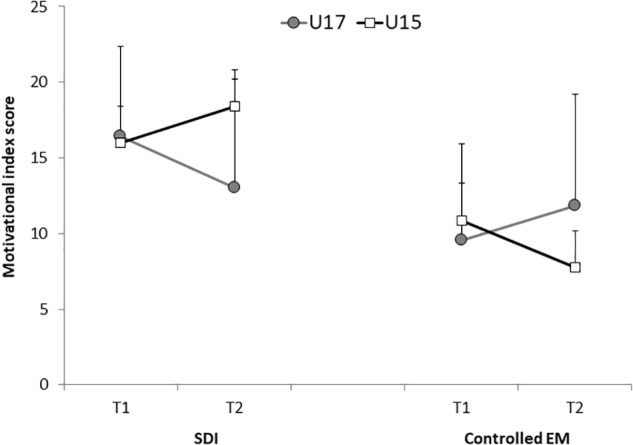
Group means (and SD bars) for global self-determined motivation (SDI) and controlled extrinsic motivation (EM) as a function of time (time 1, T1 or time 2, T2) and current (T2) age group (U15 and U17 years) for the Elite players.

Indices of motivation and data for all the behavioral regulations for T1 and T2 are shown in Table 2. For the elite groups across time, the current U15 group showed little change from T1 (U13) to T2 for autonomous motivation, whereas controlled motivation decreased (see Figure 2 for graph of controlled motivation). However, from T1 (U15) to T2 for the current, elite, U17 group, autonomous motivation showed a small decrease, whereas controlled motivation increased. There were no main effects of time for SDI or controlled motivation (both *F_s_* < 1). However, for autonomous motivation there was a tendency for an overall reduction across time, *F* (1, 29) = 4.05, *p* = 0.05, η_p_^2^ = 13, *M_difference_* = 0.70, 95% CI [0.50, 0.88]. Main effects of age category were not statistically significant for SDI, *F* (1, 29) = 2.58, *p* = 0.07, η_p_^2^ = 0.11 and controlled motivation, *F* (1, 29) = 0.89, *p* = 0.35, η_p_^2^ = 0.03. However, for autonomous motivation, the younger players (U15) scored higher than the older players (U17), *F* (1, 29) = 10.00, *p* = 0.02, η_p_^2^ = 0.26, *M_difference_* = 1.75, 95% CI [0.68, 2.82].

With respect to the more important Age × Time interactions, these were significant for SDI, *F* (1, 29) = 7.85, *p* = 0.01, η_p_^2^ = 0.21 and controlled motivation, *F* (1, 29) = 5.79, *p* = 0.02, η_p_^2^ = 0.21 (see Figure 2). However, there was no interaction for autonomous motivation, *F* (1, 29) = 1.71, *p* = 0.20, η_p_^2^ = 0.06. *Post hoc* analyses showed that for SDI, the U17s had significantly lower SDI scores than the U15s at T2 only (*p* < 0.01, *M_difference_* = 5.38, 95% CI [1.38, 9.37]) but there was no difference at T1 (*p* = 0.79). There was also a decline in SDI across time for the current U17 group (*p* = 0.05, *M_difference_* = 4.59, 95% CI [.06, 9.11]) but the increase in SDI for the U15 group (from U13 years), was not significant (*p* = 0.07). *Post hoc* analysis of controlled motivation showed significant age group differences at T2, with the now U17 group scoring higher than the now U15 group (*p* = 0.02, *M_difference_* = 4.23, 95% CI [0.72, 7.74]), but there were no group differences at T1 (*p* = 0.49). For the now U15 group, the decline in controlled motivation over time was significant (*p* = 0.02, *M_difference_* = 3.15, 95% CI [0.38, 5.92]), however, for the U17 group, the apparent increase in controlled motivation was not significant (*p* = 0.21).

#### Comparing Elite and Non-elites

When comparing the motivation indices of the elite and non-elite players, the elite groups generally scored higher than the non-elite groups (see Table 2). Separate 2 (Skill; Elite, Non-elite) × 2 (Age category; U15, U17) between groups ANOVAs supported this skill main effect for SDI, *F* (1, 61) = 13.81, *p* < 0.001, η_p_^2^ = 0.19, *M_difference_* = 4.04, 95% CI [3.65, 4.43] and autonomous motivation, *F* (1, 61) = 19.88, *p* < 0.001, η_p_^2^ = 0.25, *M_difference_* = 2.59, 95% CI [2.39, 2.79], but not controlled motivation *F* (1, 61) = 2.60, *p* = 0.16, η_p_^2^ = 0.04.

For SDI, although there was no age main effect, *F*(1, 61) = 3.57, *p* = 0.07, η_p_^2^ = 0.05, the Skill × Age interaction approached conventional levels of significance at *p* = 0.05, *F* (1, 61) = 4.29, η_p_^2^ = 0.06. For elite players, the U15 group scored significantly higher than the U17 group (*p* = 0.02, *M_difference_* = 3.90, 95% CI [3.09, 4.71]), and scored higher in comparison to the non-elite, U15 (*p* < 0.01, *M_difference_* = 5.34, 95% CI [2.66, 8.02]) and U17 groups (*p* < 0.01, *M_difference_* = 5.53, 95% CI [2.59, 8.64]). The U17 elite players were not different to the non-elite U17 (*p* = 0.17) and U15 (*p* = 0.26) groups.

For controlled motivation, the age main effect was not significant, *F* (1, 61) = 2.68, *p* = 0.10, η_p_^2^ = 0.04. Although the Skill X Age group interaction was also not significant, *F* (1, 61) = 3.65, *p* = 0.07, η_p_^2^ = 0.06, inspection of the means showed a similar trend to that for global SDI except now in the opposite direction. That is, when comparing across skill, the U15 elite players had lower scores than the U17 elites (*p* = 0.02, *M_difference_* = 1.47, 95% CI [0.62, 6.53] and the U15 (*p* < 0.05, *M_difference_* = 2.51, 95% CI [0.03, 5.34]) and U17 (*p* < 0.05, *M_difference_* = 2.51, 95% CI [0.03, 5.34]) non-elites. This was not the case for the U17 elite players, where scores were not significantly different than the non-elite, U15 (*p* = 47) and U17 (*p* = 0.65) groups. There was no age main effect for autonomous motivation, *F* (1, 61) = 2.91, *p* = 0.09, η_p_^2^ = 0.05, nor a Skill × Age interaction, *F* < 1.

### Soccer Activity Relationships With Motivation

For the elite players, neither childhood soccer practice nor play were significantly correlated with T2 indices of motivation (*r*_s_ < 0.30). Hours in organized soccer practice in the more recent 2.5 years were, however, negatively correlated with SDI (*r*_s_ = -0.59, *p* = 0.005, 95% CI [-0.77, -0.30]) and autonomous motivation (*r*_s_ = -0.52, *p* = 0.009, 95% CI [-0.74, -0.21]). Controlled motivation was moderately, positively correlated with recent soccer practice (*r*_s_ = 0.36, *p* = 0.04, 95% CI [-0.63, -0.01]). Practice hours (recent and accumulated) were not significantly related to motivation change scores (from T1 to T2) for any of the indices (*r*_s_ < 0.30). The recent hours spent in soccer play did not correlate with any of the composite measures of motivation, either for the whole sample, or for the two age groups separately.

For the non-elite players, there was a moderate, negative correlation between childhood practice and autonomous motivation (*r*_s_ = -0.35, *p* = 0.04, 95% CI [-0.62, -0.01]). This relationship was also observed for “recent” practice (*r*_s_ = -0.48, *p* = 0.03, 95% CI [-0.71, -0.16]). For SDI, there was a negative, moderate relation with recent practice (*r*_s_ = -0.40, *p* = 0.05, 95% CI [-0.66, -0.06]). As with the elite players, childhood play did not correlate with SDI, autonomous or controlled motivation in the non-elite group.

## Discussion

We tested whether measures of self-determined motivation differed as a function of age and the player’s skill and whether they were related to early practice and play experiences. Declines in SDM over time within the older (current U17) elite players were consistent with previous cross-sectional work (Hendry et al., 2014). Within the present study, older elite players exhibited a less self-determined profile at T2 (U17), including lower SDI, lower autonomous and higher controlled motivation scores, than younger elite players. These findings suggest that differences in SDM across age groups were not cohort specific (*cf.* Hendry et al., 2014), but rather are indicative of trends within elite youth soccer. The inclusion of age matched (U15, U17), non-elite soccer players provided opportunity to assess whether age related differences (or changes) in motivation were specific to these elite athletes. Elite players scored higher for SDI and autonomous motivation than the non-elites. A Skill × Age interaction for SDI showed that the younger elite (U15) participants scored significantly higher than their U17 elite counterparts and higher than both non-elite age groups, but no differences were seen across age for the non-elites. Thus, although we have data consistent with age-related differences and declines in SDM in elite athletes they were not observed for non-elite athletes. Therefore, rather than age alone being a reason for change in SDM over time, especially during adolescence as detailed in studies conducted in physical education settings (Otis et al., 2005; Barkoukis et al., 2014), differences in SDM are related to both age and skill (in elite/professional pathways in soccer). These data lead us to suspect that elite sport in general encourages or requires more SDM, which drops off around 16 years of age (U17), to levels commensurate with non-elite athletes.

The higher controlled motivation scores in the older elite players might be due to several factors. First, the proximity to the external rewards associated with professionalism (e.g., money, status) may have contributed to an increase in controlled motivation. This is consistent with meta-analytic data from education showing a shift toward more controlled forms of motivation once external rewards are introduced to previously self-determined and intrinsically rewarding activities (Deci et al., 2001). Second, the time demands placed upon elite youth athletes are vast and require an element of sacrifice from engaging in non-soccer related activities (e.g., Cook et al., 2014). Not only may this result in a sense of conflict from trying to balance sport and other activities, it may also result in a diminished sense of autonomy over their overall training schedule, which again can undermine soccer-related SDM (Pelletier et al., 2001). Although not measured within the present study, the overarching impact of the social environment within the United Kingdom Academy setting requires further consideration. Published reports have described a tendency for the motivational-climate to become more controlling with age (Partington et al., 2014), potentially impacting basic psychological needs of autonomy (Ryan and Deci, 2017).

The change scores in motivation over time were small, suggesting that the nature of the motivation remained relatively stable over this 2.5 years period (see Table 2). For the elite group, indices of autonomous motivation remained consistently high, while controlled motivation, despite increasing over time, remained relatively low (see also Zuber et al., 2014). While the elite players exhibited a largely self-determined profile, the gradual shift toward less self-determined and more controlled motivation within the older elite players hints at the emergence of co-existing forms of motivation. High scores for both autonomous and controlled motivation characterized elite fencers and runners who, despite outperforming their less elite peers, reported being more physically and emotionally exhausted (Gillet et al., 2009, 2012). An absence of a purely self-determined motivational profile is consistent with qualitative research conducted with super elite athletes (multiple gold winners at Olympic and World Championships; Hardy et al., 2017) and coach reports of former youth players that had gone on to play elite, adult soccer (e.g., Cook et al., 2014). It may be that older elite players are motivated for an innate desire for self-improvement as well as a contingent sense of self-worth attached to outperforming others (e.g., team-mates, opposition).

A second aim of this study was to test Côté and colleagues postulate that engaging in childhood play would foster later intrinsic and self-determined motivation (Côté et al., 2012). We evaluated this postulate within both an elite and a non-elite, yet competitive sample. Overall, the data did not support this postulate. There were no statistically significant (or moderately sized) relationships between indices of motivation and estimates of childhood soccer play across both samples. However, within the non-elite group, accumulated childhood practice hours were negatively related to autonomous motivation. This finding is partially in line with Côté and colleagues assertion that early practice activities may have negative psychosocial outcomes. This result is somewhat attenuated by the fact that non-elite players amassed less than half the total of childhood practice hours compared to elite players. Therefore, it is not simply the amount of soccer practice that is a concern for motivation, but perhaps it is the amount of practice invested as a function of success, or relative amounts of soccer practice (compared to other sports or play).

Recent practice amounts (practice over the last 2.5 years) were positively related to controlled motivation within the elite group and negatively associated with autonomous motivation. However, childhood soccer practice was not associated with current motivation (at T2) and change scores in motivation were not significantly associated with recent practice amounts. This suggests that factors other than practice and play were responsible for SDI change across the age groups, possibly the proximity to rewards associated with professional status.

We duly acknowledge the limitations of our approach. Retrospective recall techniques are prone to bias, yet they still remain the best method of ascertaining estimates of practice histories (see Hopwood, 2015). Because participants in the current study were still children when estimates were collected, and thus their recall would be less “retrospective” than data based on adult samples, we anticipate less of a validity issue with this method. Furthermore, a small sample of parents and coaches provided practice estimates which provided convergent validity for child estimates of soccer activity hours and the within and between questionnaire estimates for the elite players were strong and similar. Despite taking these steps, there was considerable variability between players, even at the elite levels and we acknowledge that aggregated soccer activity estimates disregard some of the subtleties associated with elite sport development, particularly at the end ranges of these practice and motivation related variables (Baker et al., 2015). Related, we acknowledge that the samples were small, creating issues for statistical power and generalization. Yet, the high level of our elite sample, allied to the prospective nature of the study and the natural attrition associated with elite soccer transitions, adds validity to our choice of sample and subsequent conclusions. Limitations are also associated with the non-elite group, given that these soccer players were from Canada, yet the elite players were from the United Kingdom. There are likely socio-cultural differences in the relative importance of soccer in these countries. While there is a thriving soccer culture in Canada, especially in locales with Major League Soccer (MLS) franchises, as was the case with the current non-elite sample, socio-cultural differences may have influenced motivation scores. That said, the non-elite players were playing at a relatively high level of competitive soccer and had participated in similar practice and play volumes to those noted in studies of United Kingdom -based recreational, yet competitive soccer players (e.g., Ford et al., 2009).

In conclusion, we have provided evidence that motivations in youth, elite soccer are dynamic and dependent on age and skill. Shifts along the OIT continuum toward less self-determined and more controlled motivation with time (and age) in elite players is likely related to the increasing competitive demands of elite youth soccer and proximity to external rewards associated with professional status (e.g., Deci et al., 2001). It does not appear to be related to an increase in hours spent in soccer activities, time or age. However, regardless of age, elite youth players were generally more autonomously motivated than the non-elite athletes. Although it is possible that childhood play activities promote enjoyment (all players participated in high volumes of childhood soccer play), there was no evidence that this early enjoyment persists in its influence with respect to enhanced SDM. Despite the lack of evidence for this key DMSP prediction, the significant negative relationship between childhood practice with autonomous motivation is partially in line with Côté and colleagues’ postulate. We suspect that these findings would generalize to other competitive situations where the necessity of high volumes of practice are required and external rewards such as government funding and professionalization are introduced to an extent that they are fundamental toward achieving elite level, adult sport status.

## Author Contributions

The manuscript is based on data collected for the Ph.D. thesis of DH. DH collected and analyzed the data and wrote versions of the manuscript. His supervisor, NH, assisted with data analysis, design and the writing. AMW and PC assisted with study ideas and writing.

## Conflict of Interest Statement

The authors declare that the research was conducted in the absence of any commercial or financial relationships that could be construed as a potential conflict of interest.
